# Spatial and Temporal Patterns of Malaria in Phu Yen Province, Vietnam, from 2005 to 2016

**DOI:** 10.4269/ajtmh.20-0392

**Published:** 2020-08-03

**Authors:** Kinley Wangdi, Sara E. Canavati, Thang Duc Ngo, Thu Minh Nguyen, Long Khanh Tran, Gerard C. Kelly, Nicholas J. Martin, Archie C. A. Clements

**Affiliations:** 1 Department of Global Health, Research School of Population Health, Australian National University, Canberra, Australia;; 2 Vysnova Partners Inc., Bethesda, Maryland;; 3 National Institute of Malariology, Parasitology, and Entomology, Hanoi, Vietnam;; 4 United States Naval Medical Research Unit Two, Phnom Penh, Cambodia;; 5 Faculty of Health Sciences, Curtin University, Bentley, Australia;; 6 Telethon Kids Institute, Nedlands, Australia

## Abstract

Malaria in Vietnam has become focal to a few provinces, including Phu Yen. This study aimed to assess correlations between intervention (population proportion protected by insecticide-treated nets and indoor residual spraying) and climatic variables with malaria incidence in Phu Yen Province. The Vietnam National Institute of Malariology, Parasitology, and Entomology provided incidence data for *Plasmodium falciparum* and *Plasmodium vivax* for 104 communes of Phu Yen Province from January 2005 to December 2016. A multivariable, zero-inflated Poisson regression model was developed with a conditional autoregressive prior structure to identify the underlying spatial structure of the data and quantify associations with covariates. There were a total of 2,778 *P. falciparum* and 1,770 *P. vivax* cases during the study period*. Plasmodium falciparum* and *P. vivax* incidence increased by 5.4% (95% credible interval [CrI] 5.1%, 5.7%) and 3.2% (95% CrI 2.9%, 3.5%) for a 10-mm increase in precipitation without lag, respectively. *Plasmodium falciparum* and *P. vivax* incidence decreased by 7.7% (95% CrI 5.6%, 9.7%) and 10.5% (95% CrI 8.3%, 12.6%) for a 1°C increase in minimum temperature without lag, respectively. There was a > 95% probability of a higher than provincial average trend of *P. falciparum* and *P. vivax* in Song Cau and Song Hoa districts. There was a > 95% probability of a lower than provincial average trend in Tuy Dong Xuan and Hoa districts for both species. Targeted distribution of resources, including intensified interventions, in this part of the province will be required for local malaria elimination.

## INTRODUCTION

Vietnam has made tremendous progress in reducing mortality and morbidity associated with malaria in recent years.^1–4^ A successful ramping-up of interventions including improvements in early and accurate diagnosis, free access to treatment with artemisinin-based combination therapies (ACTs), widespread and routine distribution of insecticide-treated mosquito nets (ITNs), and targeted and reactive indoor residual spraying (IRS) has seen a reduction in malaria cases and deaths by 97% and 99.8%, respectively, between 1991 and 2014.^3,5,6^ As a result of the significant reduction in malaria incidence, the Vietnam National Institute of Malariology, Parasitology, and Entomology (NIMPE) is pursuing an agenda of progressive elimination with a goal to eliminate local transmission by 2030.^1–4,7^

Since 1991, malaria control in Vietnam has been based on free early diagnosis and treatment with ACT, vector control through the free distribution of ITNs/long-lasting insecticidal nets (LLINs), and IRS.^8^ Quinine and chloroquine were the main treatments for *Plasmodium falciparum* and *Plasmodium vivax* until 1991. Between 1992 and 1994, artemisinin derivatives were introduced in all districts. In 1999–2000, a fixed combination of dihydroartemisinin, piperaquine, trimethoprim, and primaquine became the first-line treatment.^9^

Vector control in Vietnam underwent many changes. In 1992 and 1993, dichlorodiphenyltrichloroethane was used for IRS. Because of the rapid decline of the malaria incidence after the introduction of ITNs, IRS was largely abandoned after 1995.^8^ Since 2009, ITNs have been progressively replaced by LLINs as funds have become available through the Global Fund to Fight AIDS, Tuberculosis, and Malaria. Supplemental single LLINs or long-lasting insecticide-treated hammock nets are also now provided to mobile and migrant populations and forest-goers.^10^

In recent years, malaria has become more geographically confined to provinces in Central and Central-Southern Vietnam, including Phu Yen Province.^11^ In these areas, surges in cases have been attributed to a number of factors including the presence of exophagic and anthropophilic vectors (*Anopheles dirus*),^12,13^ barriers to control activities due to remote mountainous and forested areas,^2^ forest-related economic activities,^14–16^ and poverty.^14,17^ Furthermore, the spread of artemisinin-resistant *P. falciparum* in the Greater Mekong Subregion (GMS) poses a serious threat to malaria elimination in Vietnam.^18–22^

The aims of this study were to identify malaria clusters by species in Phu Yen Province at the commune level and assess correlations between intervention-related variables (proportion of the population protected by ITNs and IRS) and environmental variables, with malaria incidence at the commune level. The findings from this study can be used for focused interventions of malaria by the malaria program officials of Phu Yen and by malaria elimination countries.

## MATERIALS AND METHODS

### Study sites and data sources.

**Figure 1.**
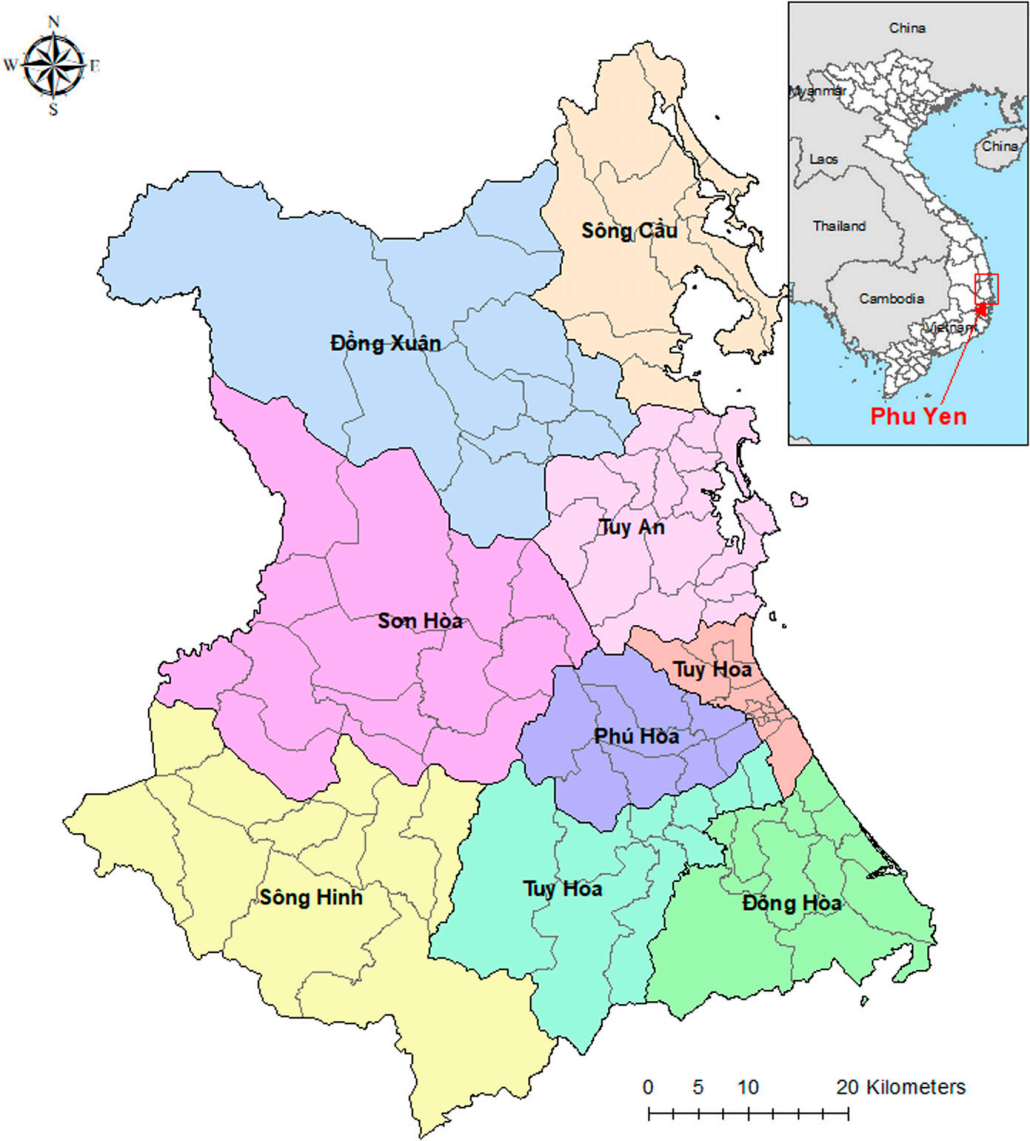
Map of Phu Yen Province in Vietnam with districts and communes. This figure appears in color at www.ajtmh.org.

Phu Yen is located in the South Central Coastal region of Vietnam. Phu Yen is administratively divided into nine districts and 104 communes (Figure 1). The total population of Phu Yen in 2016 was 875,387. Numbers of reported *P. falciparum* and *P. vivax* cases by commune and by month from January 2005 to December 2016 and ITN/IRS data were provided by the NIMPE. Commune-level population data were provided by Phu Yen provincial council. Commune population was imputed by month as follows: the difference in the district population in 2004 and 2005 was calculated and then divided by 12 to allow for a monthly population increase in 2005. A similar approach was used to calculate the monthly population of the rest of the study period (2006–2016). High-resolution (1 km^2^ [30 arc-seconds]) raster maps of interpolated long-term (1950–2000) average monthly precipitation and temperature were obtained from the WORLDCLIM website.^23^ Precipitation and temperature maps were imported into a geographical information system (GIS) (GIS; ArcMap version 10.5, ESRI, Redlands, CA) and linked spatially to a digitized boundary map of the 104 communes of Phu Yen Province. The monthly mean of precipitation and temperature were extracted for each study commune using Zonal Statistics functions in ArcMap (ESRI, Redlands, CA).

### Exploration of seasonal patterns and temporal trends.

The monthly malaria incidences by *Plasmodium* species were calculated for the full time series (January 2005–December 2016). The time series of malaria incidence was decomposed using seasonal-trend decomposition based on locally weighted regression to show the seasonal pattern, the temporal trend, and the residual variability. The time series data, the seasonal component, the trend component, and the remainder component are denoted by *Y*_*t*_, *S*_*t*_, *T*_*t*_, and *R*_*t*_, respectively, for month *t* = 1 to *N*, and

$$Y_{t}=S_{t}+T_{t}+R_{t}.$$

The parameter setting “periodic” was used for the seasonal extraction, and all other parameters were by default. In the study, logarithmic transformations were used for the time series data.^24,25^

### Crude standardized morbidity ratios (SMRs).

Crude SMRs for each commune were calculated by

$$Y_{i}=\;\frac{O_{i}}{E_{i}},$$

where *Y* is the SMR in commune *i*, *O* is the observed number of malaria cases in the commune *i*, and *E* is the expected number of malaria cases in the commune *i*, across the study period. The expected number of malaria case was calculated by multiplying the provincial malaria incidence by the average population for each commune over the entire study period.

### Independent variable selection.

Initially, a preliminary Poisson regression of total malaria cases was undertaken to select the significant covariates; of these, the best fit covariates were selected with the lowest Akaike’s information criterion (AIC). Climatic variables, namely, precipitation and minimum temperature (°C) without a lag, were selected for inclusion into the final model because these climatic variables had the best fit (Supplemental Table 1). Selected covariates for the final model were tested for collinearity, and no collinearity was found (Supplemental Table 2).

### Spatial autocorrelation analysis.

Spatial autocorrelation was explored at a global scale using Moran’s I statistic, and at a local scale estimated using the Anselin Local Moran’s I statistic (local indicators of spatial association [LISA]) and the Getis-Ord Gi* statistics. The global Moran’s I statistic was used to assess the presence and strength of spatial autocorrelation over the whole study area and to test the assumption of spatial independence in the implementation of the spatial pattern analysis. The LISA and the Getis-Ord Gi* statistics were used to detect local clustering of malaria and to identify the locations of hotspots. These analyses were conducted using tools provided in ArcMap software.^26^

### Spatiotemporal model.

Of the 14,976 observations, there were 13,350 (89.1%) zero counts for *P. falciparum* and 13,864 (92.6%) zero counts for *P. vivax*. Zero counts can arise from two processes: “excess zeros” (also called structural zeros), for which the process of their occurrence is different from the “random zeros” that arise as part of the Poisson process that generates the malaria counts. One possible explanation for the excess zeros is that they arise in communes that were unable to support malaria transmission during the study period for a variety of epidemiological reasons such as vector habitat unsuitability or isolation from areas with ongoing transmission. Zero-inflated Poisson (ZIP) regression was a better model with lower AIC and Bayesian Information Criterion (BIC) than the standard Poisson regression, and the Vuong test showed the two models were statically different (Supplemental Table 3). Bayesian statistical software WinBUGS version 1.4 (Medical Research Council, Cambridge, United Kingdom, and Imperial College London, London, United Kingdom) was used to develop ZIP regression models for *P. falciparum* and *P. vivax* separately. They contained a mixing probability ω that the observation is an excess zero count. The model included climatic variables (minimum temperature and precipitation); proportion of the population covered by ITNs and IRS, as explanatory variables; and spatially structured and unstructured random effects.

For the count of malaria cases *Y*, in the *i*th commune (*i* = 1…104) and the *j*th month (January 2005–December 2016), the model was structured as follows:

$$P\left(Y_{ij}=\;y_{ij}\right)=\{\begin{array}{cc} \omega +1\;\left(1-\omega \right)e^{-\mu },\; & \;y_{ij}=0\\ \left(1-\omega \right)e^{-\mu }\;\mu_{ij}^{y_{ij}}/y_{ij}, & y_{ij}>0; \end{array}$$

$$Y_{ij}∼\text{Poisson }\left(\mu_{ij}\right),$$

$$\text{log }\left(\mu_{\mathrm{ij}}\right)\;=\text{ log}\left(E_{\mathrm{ij}}\right)+\theta_{\mathrm{ij}},$$

$$\theta_{\mathrm{ij}}\;=\;\alpha \;+\;\beta_{1}{\text{× Pop protected}}_{\mathrm{ij}}\;+\;\beta_{2}{\text{ × Precipitation}}_{\mathrm{ij}}\;+\;\beta_{3}{\text{ × Tmin}}_{\mathrm{ij}}\;+\;\beta_{4}{\text{ × trend}}_{j}\;+\;u_{i}\;+\;s_{i}\;+\;w_{\mathrm{ij}},$$

where *E*_*ij*_ is the expected number of cases (acting as an offset to control for population size) in commune *i* and month *j*, and θ_*ij*_ is the mean log relative risk (RR); α is the intercept; and β_1_, β_2_, β_3_, and β_4_ are the coefficients of proportion of population covered by ITNs and IRS, the overall temporal trend of malaria precipitation and minimum temperature; unstructured, spatially structured, and spatiotemporal random effect were denoted by *u*_*i*_, *s*_*i*_, and *w*_*ij*_ which assumed to a variance σ_s_^2^ and mean of zero.

A conditional autoregressive prior structure was used to model the spatially structured random effect. Spatial relationships between the communes were determined using a queen contiguity. For two communes sharing a border, an adjacency weight of 1 was assigned, whereas if they did not, the weight was 0. An unbounded uniform (i.e., flat) prior distribution was specified for the intercept, whereas a non-informative normal prior distribution (i.e., with a wide variance, σ^2^ = 1,000) was used for the coefficients. The priors for the precision of unstructured and spatially structured random effects (1/σ_u_^2^ and 1/σ_s_^2^) were specified using non-informative gamma distributions, with shape and scale parameters equal to 0.01.

An initial 10,000 burn-in iterations were discarded. Convergence was examined by running the subsequent blocks of 20,000 iterations, by visual inspection of posterior density and history plots, and occurred at approximately 100,000 iterations for each model. The posterior distributions of each model’s parameters were stored after the convergence (100,000 iterations). The summary of the analysis was performed with the posterior mean and 95% credible intervals (CrIs). In all analyses, an α-level of 0.05 was adopted to indicate statistical significance (as indicated by 95% CrI for RR that excluded 1). ArcMap 10.5.1 software (ESRI, Redlands, CA) was used to generate the maps of spatial distribution of posterior means of the unstructured and structured random effects obtained from the three models.

## RESULTS

### Descriptive analysis.

**Figure 2.**
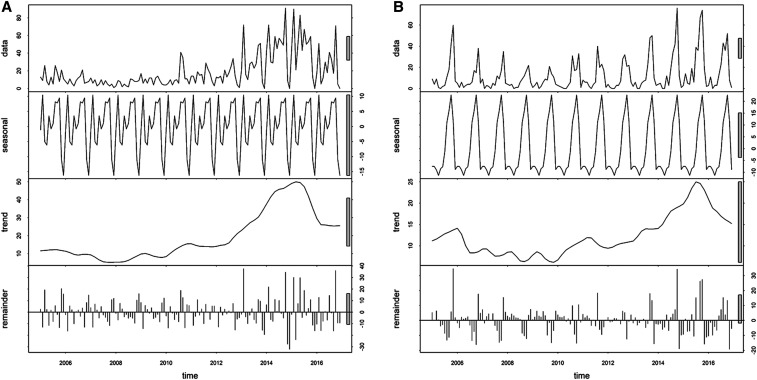
Decomposed monthly (**A**) *Plasmodium falciparum* and (**B**) *Plasmodium vivax* incidence in Phu Yen Province, Vietnam, 2005–2016.

There were 2,778 *P. falciparum* and 1,770 *P. vivax* cases during the study period. The proportion of *P. falciparum* cases continued to decrease from 79.0% (211) in 2005 to 50.0% (42) in 2016, whereas the proportion of *P. vivax* increased from 21.0% (55) to 50.0% (42) during the same period. The annual parasite incidence for the study period was 0.28 and 0.18 cases per 1,000 person-years at risk for *P. falciparum* and *P. vivax*, respectively (Table 1). Both species of malaria displayed a strong seasonal pattern, with incidence increases starting in September and peaking in November (Figure 2, Supplemental Figure 1). Both were heterogeneously distributed across the province, with high SMRs in Dong Xuan and Song Hon districts (Supplemental Figure 2).

**Table 1:** Malaria incidence during the study period (2005–2016)

Year	Population	*Plasmodium falciparum*			*Plasmodium vivax*		
		Cases	Proportion of total cases	API	Cases	Proportion of total cases	API
2005	784,003	211	0.79	0.27	55	0.21	0.07
2006	791,922	352	0.86	0.44	56	0.14	0.07
2007	799,921	168	0.81	0.21	40	0.19	0.05
2008	808,001	156	0.92	0.19	13	0.08	0.02
2009	816,163	274	0.85	0.34	48	0.15	0.06
2010	824,407	222	0.80	0.27	57	0.20	0.07
2011	832,734	244	0.65	0.29	133	0.35	0.16
2012	841,146	331	0.54	0.39	281	0.46	0.33
2013	849,642	327	0.49	0.38	339	0.51	0.40
2014	858,138	348	0.45	0.41	419	0.55	0.49
2015	866,720	103	0.26	0.12	287	0.74	0.33
2016	875,387	42	0.50	0.05	42	0.50	0.05
Overall	9,948,183	2,778	0.61	0.28	1,770	0.39	0.18

API = annual parasite incidence.

### Malaria clusters.

**Figure 3.**
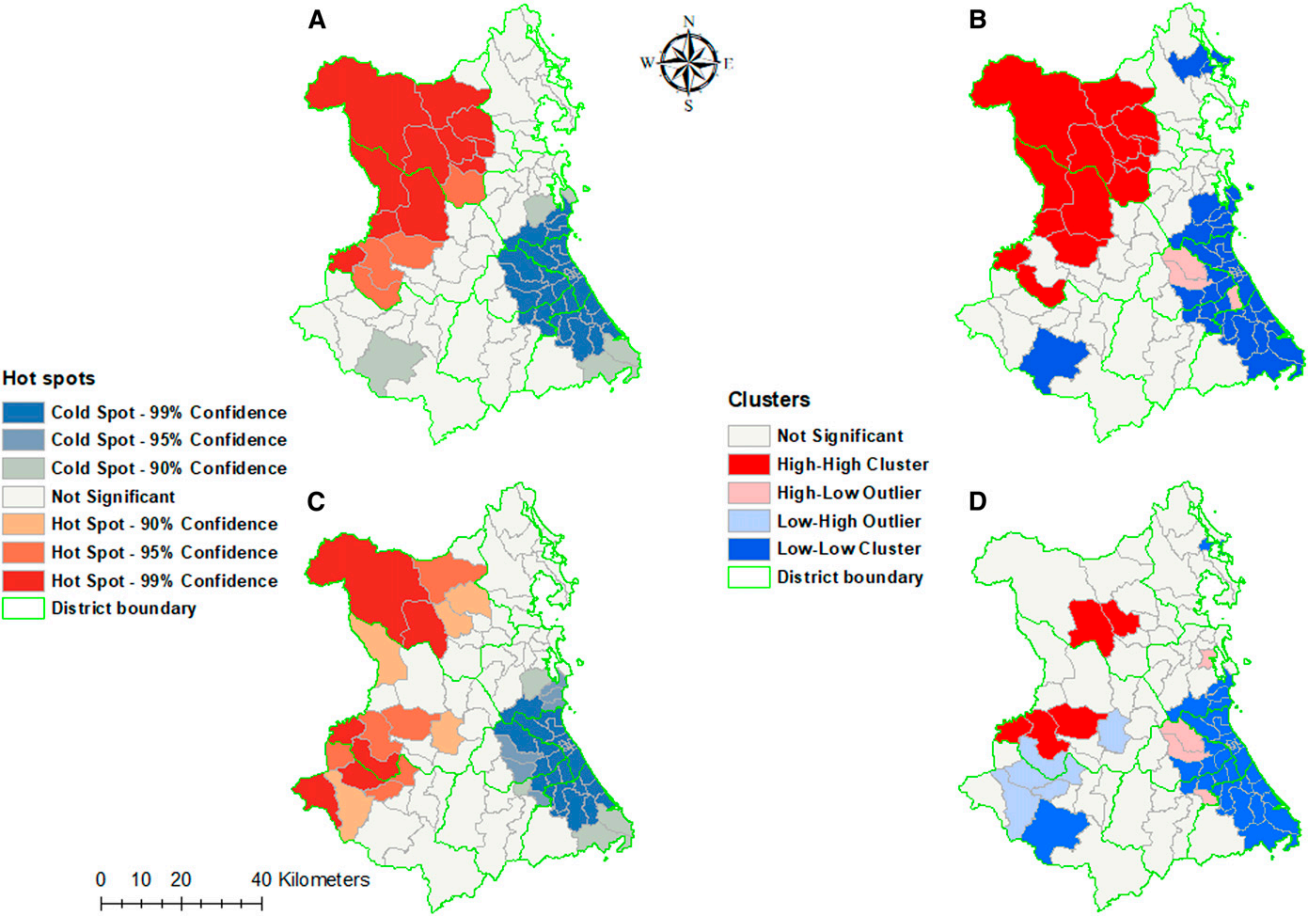
Malaria cluster maps by communes of Phu Yen Province, Vietnam, 2005–2016. (**A**) Getis-Ord Gi* statistics and (**B**) Anselin’s Local Moran’s I for *Plasmodium falciparum*. (**C**) Getis-Ord Gi* statistics and (**D**) Anselin’s Local Moran’s I for *Plasmodium vivax*. This figure appears in color at www.ajtmh.org.

The Global Moran’s I showed significant spatial autocorrelation for both *P. falciparum* (*z* score = 9.30; *P* < 0.0001) and *P. vivax* (*z* score = 5.94; *P* < 0.0001) (Supplemental Figures 3 and 4). Hotspot analysis using the Getis-Ord Gi* statistic showed that 13 hotspots for *P. falciparum* and 11 hotspots *P. vivax* were located in the communes of Dong Xuan and Son Hoa districts, whereas 37 *P. falciparum* and 35 *P. vivax* coldspots were located in Dong Hoa, Phu Hoa, and Tuy Hoa districts. Nevertheless, cluster analysis using LISA showed only 18 *P. falciparum* and 5 *P. vivax* high–high clusters in Dong Xuan, Phu Hoa, and Son Hoa districts (Figure 3).

### Spatiotemporal model.

*Plasmodium falciparum* incidence decreased by 6.6% (95% CrI 0.6%, 13.3%) and *P. vivax* incidence increased by 89% (95% CrI 72.5%, 107.1%) every month during the study period. A 10-mm increase in precipitation was associated with an increase in *P. falciparum* and *P. vivax* by 5.4% (95% CrI 5.1%, 5.7%) and 3.2% (95% CrI 2.9%, 3.5%), respectively. A minimum temperature increase of 1°C was associated with a decrease in *P. falciparum* and *P. vivax* risk of 7.7% (95% CrI 5.6%, 9.7%) and 10.5% (95% CrI 8.3%, 12.6%), respectively. The model showed that every 10% increase in population protected by IRS and ITNs was associated with a decrease in incidence of *P. falciparum* by 11%. However, these decreases were not statistically significant (Table 2).

**Table 2:** Regression coefficients and 95% CrI from Bayesian spatial and nonspatial models of *P. falciparum* and *P. vivax* cases reported by month and communes in Phu Yen Province, Vietnam, 2005–2016

Variable	*P. falciparum* RR (95% CrI)	*P. vivax* RR (95% CrI)
Intercept*	−1.14 (−1.38, −0.94)	−1.23 (−1.48, −1.02)
Population protected (10% increase)†	0.999 (0.998, 1.00)	1.00 (0.998, 1.001)
Precipitation (10 mm increase)	1.054 (1.051, 1.057)	1.032 (1.029, 1.035)
Temperature minimum (°Celsius)	0.923 (0.903, 0.944)	0.895 (0.874, 0.917)
Mean monthly trend	0.934 (0.867, 1.006)	1.89 (1.725, 2.071)
Proportion of zero	0.218 (0.171, 0.264)	0.277 (0.226, 0.327)
Heterogeneity		
Unstructured	2.537 (0.689, 8.063)	2.038 (0.581, 6.879)
Structured (spatial)	0.153 (0.091, 0.258)	0.155 (0.084, 0.283)
Structured (trend)	2.903 (1.681, 4.675)	2.45 (1.364, 4.084)

CrI = credible interval; *P. falciparum = Plasmodium falciparum*; *P. vivax = Plasmodium vivax*; RR = relative risk.

* Coefficients.

† Proportion of population protected by preventive measures.

**Figure 4.**
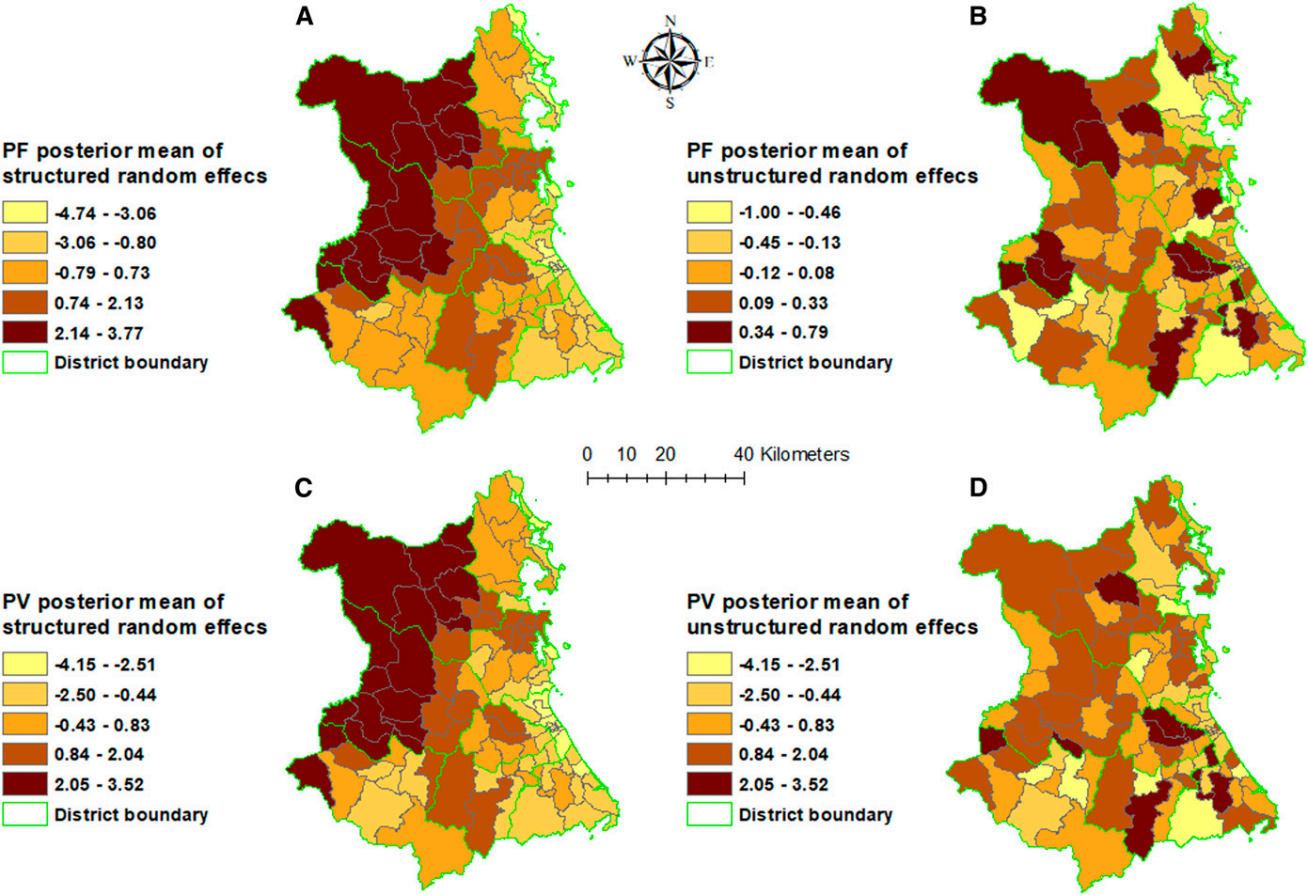
Spatial distribution of the posterior means of structured and unstructured random effects from model in Phu Yen Province, Vietnam, 2005–2016. (**A**) Structured and (**B**) unstructured random effects of *Plasmodium falciparum*; (**C**) structured and (**D**) unstructured random effects of *Plasmodium vivax*. This figure appears in color at www.ajtmh.org.

The spatially auto-correlated random effect (*v*_*i*_) smooths the spatial pattern of residual variation in malaria incidence after taking into account the fixed effects (Figure 4). Both types of malaria showed areas of lower than average residual malaria risk in Song Cau, Tuy An, Phu Hoa, Dong Hoa, and Tuy Hoa districts. For both types of malaria, areas of higher than average residual malaria risk were found in Dong Xuan, Son Hoa, and Song Hinh districts.

There was > 95% probability of a higher than provincial average trend of *P. falciparum* in 10/104 communes, which were mostly located in the Son Hoa districts. Similarly, 11/104 communes had > 95% probability of a higher than national average increasing trend of *P. vivax*, also mostly located in Song Cau and Song Hoa districts. For both *P. falciparum* and *P. vivax*, 15/104 districts had > 95% probability of a trend less than the provincial average, mostly located in Tuy Dong Xuan and Hoa districts (Supplemental Figure 5).

## DISCUSSION

Using a surveillance dataset of 12 years (2005–2016), the present study has demonstrated substantial changes occurring with respect to annual trends and the geographical distribution of malaria in Phu Yen Province. In this study, we found that malaria hotspots for both species were found in Dong Xuan and Son Hoa districts. *Plasmodium falciparum* trend decreased, whereas *P. vivax* showed an increasing trend. Both species of malaria displayed a strong seasonal pattern. Prevention measures including LLINs and IRS were not significant predictors of malaria incidence. Minimum temperature was associated with reduction in malaria incidence, whereas precipitation was associated with increase in malaria incidence.

Malaria cases showed a strong seasonal pattern, with cases increasing from September and peaking in November each year. This pattern was associated with the rainy season in Phu Yen, where two seasons (dry, from January to August, and rainy, from September to December) were distinguished. Other studies reported a similar association with rainfall in Bhutan, India, and other parts of the world.^27–35^ This finding is consistent with that of the published literature from Vietnam.^17,36^ During the study period, *P. falciparum* showed a decreasing trend, whereas the opposite was true for *P. vivax*, which is similar to the national trend.^37^ As countries embark on malaria elimination, *P. falciparum* incidence declines more rapidly than the incidence of *P. vivax* because of the greater effectiveness of vector control interventions on the former.^38^ Treating all stages of the parasite (radical cure) is a critical strategy for the successful control and ultimate elimination of *P. vivax*.^39^

Malaria risk continues to decline in Vietnam, and transmission is becoming increasingly heterogeneous, with most cases now concentrated within a relatively small number of communes, including Phu Yen.^11^ Such patterns are consistent with those of other countries with low malaria burden.^27,35,40–43^ Therefore, spatially targeting interventions and associated resources are likely to achieve better results than a uniform approach to the distribution and delivery of malaria reduction interventions.^44–46^ Geographical information system–based spatial decision support systems (SDSSs) are one of the tools currently being used in countries in the Asia-Pacific region to support enhanced surveillance in priority areas, primarily as a means for locating malaria transmission, identifying and targeting appropriate foci-specific interventions, and ensuring these interventions are implemented at optimal levels of coverage.^47–49^ Combining the capacity to identify priority malaria clusters and associated appropriate foci-specific interventions with enhanced surveillance and intervention management tools such as a SDSS provides significant potential opportunity to target and strengthen elimination efforts where needed.^45,50^

This study found evidence of significant spatial variability in malaria incidence within Phu Yen Province. This likely arose because of two main processes: first, the effects of the covariates in the model (preventive coverage and climate) and, second, the residual effects of additional, unmeasured influences on malaria incidence that were captured by the random effects—these were both spatially structured and unstructured. Given that the areas of high residual risk were in the western part of the province, proximity to other high-transmission areas might partly explain this residual variation; however, further investigation is required.

One of the strengths of this study was the capacity to implement the spatial analysis at a relatively fine resolution, being the commune level. Traditionally, spatial patterns of malaria risk have been displayed at larger geographical units, such as at district, province, national, regional, and global scales.^51,52^ However, such resolution may mask more localized underlying patterns of disease through averaging.^53^ Therefore, use of finer geographic units such as communes may be necessary to observe important local variation in spatial patterns of malaria risk and to better guide disease control efforts and resource allocation, particularly when transmission declines to levels favorable to the pursuit of elimination.^44^

This study used Bayesian statistical methods to quantify seasonal and commune variations of *P. falciparum* and *P. vivax* and the effects of climatic factors. The finding from this analysis indicated that precipitation and temperature were important drivers of spatiotemporal patterns of malaria incidence in Phu Yen. Although there was a significant reduction in malaria incidence, this success has not been evenly distributed throughout Phu Yen, and spatial heterogeneities remain (Figure 4). Targeted distribution of resources should be implemented using evidence-based approaches, supported by spatiotemporal analytical methods, to assist more effective malaria control in Phu Yen Province where these resources are most needed.

A limitation of the study included the use of routine case reports to measure incidence. Known issues exist surrounding completeness and representativeness of such data. It has been reported that routine reporting of malaria cases through the health information system in Vietnam underestimates the true number of cases.^54^ Whether these factors affect the validity of our analysis depends on whether or not underreporting systematically differs between communes and time-periods. Second, populations of districts were projected and may have led to over or under estimation. Third, we used long-term interpolated climatic variables because there were no data that coincided with the study time period. This might have impacted the spatiotemporal distribution of malaria. Finally, unmeasured risk modifiers, such as socioeconomic development, living standards, treatment, localized behavioral patterns, population mobility, imported cases, and bed net use, are unaccounted for in this study.^55–57^

## CONCLUSION

Minimum temperature was associated with decreased risk, whereas precipitation was associated with increased risk of both *P. falciparum* and *P. vivax*. A high residual risk area of malaria transmission (after accounting for intervention and climate variables) was identified in the northwestern part of Phu Yen Province. Hence, targeted distribution of resources, including intensified interventions, in this part of the province will be required for local malaria elimination. Similar approaches can be used for identifying spatial heterogeneity of malaria transmission for resource allocation by malaria elimination countries.

## Supplemental figures and tables

Supplemental materials

## References

[1] MoH, 2011 National Strategy for Malaria Control and Elimination for the Period of 2012–2015*.*

[2] Hung le Q Vries PJ Giao PT Nam NV Binh T, 2002 Control of malaria: a successful experience from Vietnam. Bull World Health Organ 80: 660–666. 12219158 PMC2567582

[3] Barat LM, 2006 Four malaria success stories: how malaria burden was successfully reduced in Brazil, Eritrea, India, and Vietnam. Am J Trop Med Hyg 74: 12–16. 16407339

[4] Morrow M Nguyen QA Caruana S Biggs BA Doan NH Nong TT, 2009 Pathways to malaria persistence in remote central Vietnam: a mixed-method study of health care and the community. BMC Public Health 9: 85. 19309519 10.1186/1471-2458-9-85 PMC2666724

[5] Thang ND Erhart A Hung le X Thuan LK Xa NX Thanh NN Van Ky P Coosemans M Speybroeck N D’Alessandro U, 2009 Rapid decrease of malaria morbidity following the introduction of community-based monitoring in a rural area of central Vietnam. Malar J 8: 3. 19123932 10.1186/1475-2875-8-3 PMC2657912

[6] World Health Organization, 2014 Malaria: Fact Sheet. Available at: http://www.wpro.who.int/vietnam/topics/malaria/factsheet/en/. Accessed August 30, 2017.

[7] WHO, 2015 Strategy for Malaria Elimination in the Greater Mekong Subregion: 2015–2030. Geneva, Switzerland: World Health Organization.

[8] Nam NV de Vries PJ Toi VL Nagelkerke N, 2005 Malaria control in Vietnam: the Binh Thuan experience. Trop Med Int Health 10: 357–365. 15807800 10.1111/j.1365-3156.2005.01387.x

[9] Giao PT de Vries PJ Hung le Q Binh TQ Nam NV Kager PA, 2004 CV8, a new combination of dihydroartemisinin, piperaquine, trimethoprim and primaquine, compared with atovaquone-proguanil against falciparum malaria in Vietnam. Trop Med Int Health 9: 209–216. 15040557 10.1046/j.1365-3156.2003.01180.x

[10] WPRO, 2018 National Malaria Programme Review – Viet Nam. Manila, Philippines.

[11] Thanh PV, 2015 Epidemiology of forest malaria in central Vietnam: the hidden parasite reservoir. Malar J 14: 86. 25880664 10.1186/s12936-015-0601-y PMC4342195

[12] Manh CD Beebe NW Van VN Quang TL Lein CT Nguyen DV Xuan TN Ngoc AL Cooper RD, 2010 Vectors and malaria transmission in deforested, rural communities in north-central Vietnam. Malar J 9: 259. 20846447 10.1186/1475-2875-9-259 PMC2945362

[13] Trung HD Bortel WV Sochantha T Keokenchanh K Briet OJ Coosemans M, 2005 Behavioural heterogeneity of *Anopheles* species in ecologically different localities in Southeast Asia: a challenge for vector control. Trop Med Int Health 10: 251–262. 15730510 10.1111/j.1365-3156.2004.01378.x

[14] Erhart A 2005 Epidemiology of forest malaria in central Vietnam: a large scale cross-sectional survey. Malar J 4: 58. 16336671 10.1186/1475-2875-4-58 PMC1325238

[15] Erhart A Thang ND Hung NQ Toi LV Hung LX Tuy TQ Cong LD Speybroeck N Coosemans M D’Alessandro U, 2004 Forest malaria in Vietnam: a challenge for control. Am J Trop Med Hyg 70: 110–118. 14993619

[16] Grietens KP Xuan XN Ribera J Duc TN van Bortel W Ba NT Van Ky P Xuan HL D’Alessandro U Erhart A, 2012 Social determinants of long lasting insecticidal hammock use among the Ra-glai ethnic minority in Vietnam: implications for forest malaria control. PLoS One 7: e29991. 22253852 10.1371/journal.pone.0029991 PMC3257264

[17] Bui HM 2011 Social and environmental determinants of malaria in space and time in Viet Nam. Int J Parasitol 41: 109–116. 20833173 10.1016/j.ijpara.2010.08.005 PMC3086784

[18] Imwong M 2017 The spread of artemisinin-resistant *Plasmodium falciparum* in the Greater Mekong subregion: a molecular epidemiology observational study. Lancet Infect Dis 17: 491–497. 28161569 10.1016/S1473-3099(17)30048-8 PMC5406483

[19] Hien TT 2012 In vivo susceptibility of *Plasmodium falciparum* to artesunate in Binh Phuoc province, Vietnam. Malar J 11: 355. 23101492 10.1186/1475-2875-11-355 PMC3504531

[20] World Health Organization, 2016 World Malaria Report 2016. WHO Library Cataloguing-in-Publication Data.

[21] World Health Organization, 2010 Global Report on Antimalarial Drug Efficacy and Drug Resistance: 2000–2010*.* WHO Library Cataloguing-in-Publication Data.

[22] Noedl H Se Y Schaecher K Smith BL Socheat D Fukuda MM, 2008 Evidence of artemisinin-resistant malaria in western Cambodia. N Engl J Med 359: 2619–2620. 19064625 10.1056/NEJMc0805011

[23] Worldclim, 2017 Worldclim - Global Climate Data. Free Climate Data for Ecological Modeling and GIS.

[24] Cleveland RB, 1990 STL: a seasonal-trend decomposition procedure based on loess. J Offic Stat.

[25] Childs DZ Cattadori IM Suwonkerd W Prajakwong S Boots M, 2006 Spatiotemporal patterns of malaria incidence in northern Thailand. Trans R Soc Trop Med Hyg 100: 623–631. 16406037 10.1016/j.trstmh.2005.09.011

[26] Wong DWS Lee J, 2005 Statistical Analysis of Geographic Information with ArcView GIS and Arc GIS*.*

[27] Wangdi K Kaewkungwal J Singhasivanon P Silawan T Lawpoolsri S White NJ, 2011 Spatio-temporal patterns of malaria infection in Bhutan: a country embarking on malaria elimination. Malar J 10: 89. 21496285 10.1186/1475-2875-10-89 PMC3094227

[28] Dev V Phookan S Sharma VP Anand SP, 2004 Physiographic and entomologic risk factors of malaria in Assam, India. Am J Trop Med Hyg 71: 451–456. 15516642

[29] Dev V Phookan S Sharma VP Dash AP Anand SP, 2006 Malaria parasite burden and treatment seeking behavior in ethnic communities of Assam, Northeastern India. J Infect 52: 131–139. 16442438 10.1016/j.jinf.2005.02.033

[30] Sharma PK Ramakrishnan R Hutin YJ Gupte MD, 2009 Increasing incidence of malaria in Kurseong, Darjeeling district, West Bengal, India, 2000–2004. Trans R Soc Trop Med Hyg 103: 691–697. 18786685 10.1016/j.trstmh.2008.07.019

[31] Wardrop NA Barnett AG Atkinson JA Clements AC, 2013 *Plasmodium vivax* malaria incidence over time and its association with temperature and rainfall in four counties of Yunnan Province, China. Malar J 12: 452. 24350670 10.1186/1475-2875-12-452 PMC3878361

[32] Zhao X Chen F Feng Z Li X Zhou XH, 2014 The temporal lagged association between meteorological factors and malaria in 30 counties in south-west China: a multilevel distributed lag non-linear analysis. Malar J 13: 57. 24528891 10.1186/1475-2875-13-57 PMC3932312

[33] Shapiro LLM Whitehead SA Thomas MB, 2017 Quantifying the effects of temperature on mosquito and parasite traits that determine the transmission potential of human malaria. PLoS Biol 15: e2003489. 29036170 10.1371/journal.pbio.2003489 PMC5658182

[34] Teklehaimanot HD Lipsitch M Teklehaimanot A Schwartz J, 2004 Weather-based prediction of *Plasmodium falciparum* malaria in epidemic-prone regions of Ethiopia I. Patterns of lagged weather effects reflect biological mechanisms. Malar J 3: 41. 15541174 10.1186/1475-2875-3-41 PMC535540

[35] Wangdi K Xu Z Suwannatrai AT Kurscheid J Lal A Namgay R Glass K Gray DJ Clements ACA, 2020 A spatio-temporal analysis to identify the drivers of malaria transmission in Bhutan. Sci Rep 10: 7060. 32341415 10.1038/s41598-020-63896-7 PMC7184595

[36] Paaijmans KP Blanford S Bell AS Blanford JI Read AF Thomas MB, 2010 Influence of climate on malaria transmission depends on daily temperature variation. Proc Natl Acad Sci U S A 107: 15135–15139. 20696913 10.1073/pnas.1006422107 PMC2930540

[37] Wangdi K Canavati SE Ngo TD Tran LK Nguyen TM Tran DT Martin NJ Clements ACA, 2018 Analysis of clinical malaria disease patterns and trends in Vietnam 2009–2015. Malar J 17: 332. 30223843 10.1186/s12936-018-2478-z PMC6142383

[38] World Health Organization, 2015 Confronting Plasmodium vivax Malaria.

[39] Bassat Q Velarde M Mueller I Lin J Leslie T Wongsrichanalai C Baird JK, 2016 Key knowledge gaps for *Plasmodium vivax* control and elimination. Am J Trop Med Hyg 95: 62–71. 27430544 10.4269/ajtmh.16-0180 PMC5201224

[40] Bousema T Griffin JT Sauerwein RW Smith DL Churcher TS Takken W Ghani A Drakeley C Gosling R, 2012 Hitting hotspots: spatial targeting of malaria for control and elimination. PLoS Med 9: e1001165. 22303287 10.1371/journal.pmed.1001165 PMC3269430

[41] Rosas-Aguirre A Ponce OJ Carrasco-Escobar G Speybroeck N Contreras-Mancilla J Gamboa D Pozo E Herrera S Llanos-Cuentas A, 2015 *Plasmodium vivax* malaria at households: spatial clustering and risk factors in a low endemicity urban area of the northwestern Peruvian coast. Malar J 14: 176. 25903826 10.1186/s12936-015-0670-y PMC4416302

[42] Rulisa S Kateera F Bizimana JP Agaba S Dukuzumuremyi J Baas L Harelimana JDD Mens PF Boer KR de Vries PJ, 2013 Malaria prevalence, spatial clustering and risk factors in a low endemic area of eastern Rwanda: a cross sectional study. PLoS One 8: e69443. 23936018 10.1371/journal.pone.0069443 PMC3720654

[43] Wangdi K Gatton ML Kelly GC Clements AC, 2015 Cross-border malaria: a major obstacle for malaria elimination. Adv Parasitol 89: 79–107. 26003036 10.1016/bs.apar.2015.04.002

[44] Clements ACA Reid HL Kelly GC Hay SI, 2013 Further shrinking the malaria map: how can geospatial science help to achieve malaria elimination? Lancet Infect Dis 13: 709–718. 23886334 10.1016/S1473-3099(13)70140-3

[45] Wangdi K Clements ACA, 2018 Ending Malaria Transmission in the Asia Pacific Malaria Elimination Network (APMEN) Countries: Challenges and the Way Forward. IntechOpen.

[46] Wangdi K Banwell C Gatton ML Kelly GC Namgay R Clements AC, 2016 Malaria burden and costs of intensified control in Bhutan, 2006–14: an observational study and situation analysis. Lancet Glob Health 4: e336–e343. 27102197 10.1016/S2214-109X(16)00083-8

[47] Wangdi K Banwell C Gatton ML Kelly GC Namgay R Clements AC, 2016 Development and evaluation of a spatial decision support system for malaria elimination in Bhutan. Malar J 15: 180. 27004465 10.1186/s12936-016-1235-4 PMC4804570

[48] Kelly GC Seng CM Donald W Taleo G Nausien J Batarii W Iata H Tanner M Vestergaard LS Clements ACA, 2011 A spatial decision support system for guiding focal indoor residual spraying interventions in a malaria elimination zone. Geospatial Health 6: 21–31. 22109860 10.4081/gh.2011.154

[49] Ngo TD Canavati SE Dinh HS Ngo TD Tran DT Martin NJ Kelly GC, 2019 Addressing operational challenges of combatting malaria in a remote forest area of Vietnam using spatial decision support system approaches. Geospat Health 14. 10.4081/gh.2019.770 31724368

[50] Kelly GC Hii J Batarii W Donald W Hale E Nausien J Pontifex S Vallely A Tanner M Clements A, 2010 Modern geographical reconnaissance of target populations in malaria elimination zones. Malar J 9: 289. 20961423 10.1186/1475-2875-9-289 PMC2974750

[51] Clements AC Barnett AG Cheng ZW Snow RW Zhou HN, 2009 Space-time variation of malaria incidence in Yunnan province, China. Malar J 8: 180. 19646240 10.1186/1475-2875-8-180 PMC2724544

[52] Hundessa SH Williams G Li S Guo J Chen L Zhang W Guo Y, 2016 Spatial and space-time distribution of *Plasmodium vivax* and *Plasmodium falciparum* malaria in China, 2005–2014. Malar J 15: 595. 27993171 10.1186/s12936-016-1646-2 PMC5168843

[53] Haddow AD Jones CJ Odoi A, 2009 Assessing risk in focal arboviral infections: are we missing the big or little picture? PLoS One 4: e6954. 19742311 10.1371/journal.pone.0006954 PMC2734166

[54] Erhart A 2007 Accuracy of the health information system on malaria surveillance in Vietnam. Trans R Soc Trop Med Hyg 101: 216–225. 16979202 10.1016/j.trstmh.2006.07.003

[55] Goesch J 2008 Socio-economic status is inversely related to bed net use in Gabon. Malar J 7: 60. 18423025 10.1186/1475-2875-7-60 PMC2358918

[56] Njau JD Stephenson R Menon M Kachur SP McFarland DA, 2013 Exploring the impact of targeted distribution of free bed nets on households bed net ownership, socio-economic disparities and childhood malaria infection rates: analysis of national malaria survey data from three sub-Saharan Africa countries. Malar J 12: 245. 23855893 10.1186/1475-2875-12-245 PMC3720242

[57] Wangdi K Gatton M Kelly G Clements A, 2014 Prevalence of asymptomatic malaria and bed net ownership and use in Bhutan, 2013: a country earmarked for malaria elimination. Malar J 13: 352. 25190579 10.1186/1475-2875-13-352 PMC4161830

