# Supplementary material for: Spatial and Temporal Patterns of Malaria in Phu Yen Province, Vietnam, from 2005 to 2016
Source: Am J Trop Med Hyg. 2020 Aug 3;103(4):1540–8. doi: 10.4269/ajtmh.20-0392 (PMC7543816; doi:10.4269/ajtmh.20-0392)
Supplement: Supplementary file 1 [file tpmd200392.SD1.pdf]

Supplementary materials

Supplementary Figure 1 Time series of numbers of reported cases of malaria due to Plasmodium falciparum and Plasmodium vivax, Phu Yen, 2005-2016.

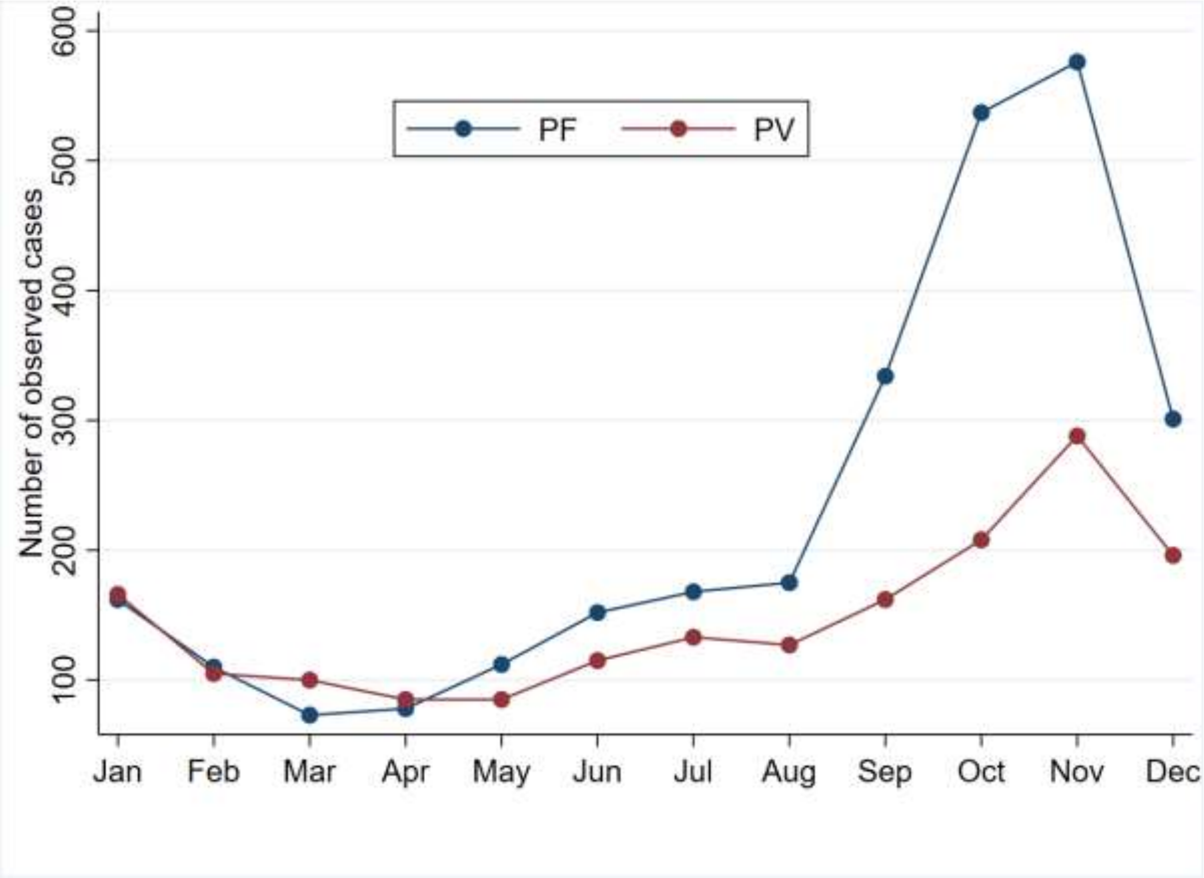

Supplementary Figure 2 Crude standardized morbidity ratios (SMR) of (a) *Plasmodium falciparum* (b) *Plasmodium vivax* by communes of Phu Yen Province, Viet Nam, 2005-2016.

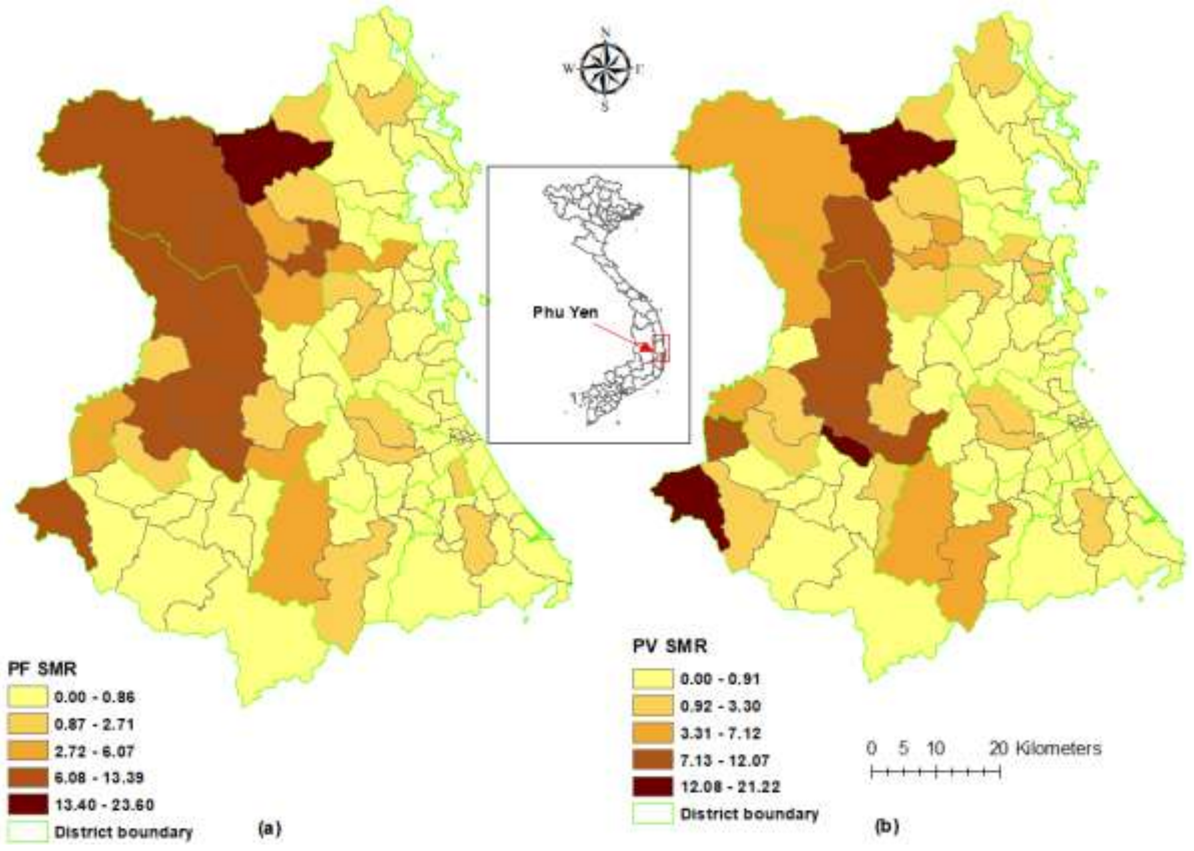

Supplementary Figure 3 Spatial Autocorrelation Report for *Plasmodium falciparum*

**Moran's Index:**	0.209888
**z-score:**	9.296555
**p-value:**	0.000000

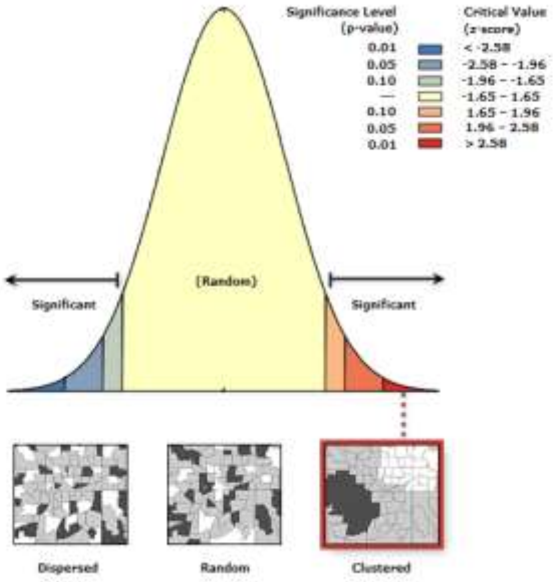

Given the z-score of 9.296555, there is a less than 1% likelihood that this clustered pattern could be the result of random chance.

Global Moran's I Summary	
**Moran's Index:**	0.209888
**Expected Index:**	-0.009709
**Variance:**	0.000558
**z-score:**	9.296555
**p-value:**	0.000000

Dataset Information	
**Input Feature Class:**	Phu_yen_commune
**Input Field:**	PF_CASES.CSV.INC
**Conceptualization:**	INVERSE_DISTANCE
**Distance Method:**	EUCLIDEAN
**Row Standardization:**	False
**Distance Threshold:**	17931.5800 Meters
**Weights Matrix File:**	None
**Selection Set:**	False

Supplementary Figure 4 Spatial Autocorrelation Report for *Plasmodium vivax*

Moran's Index: 0.131331
z-score: 5.936701
p-value: 0.000000

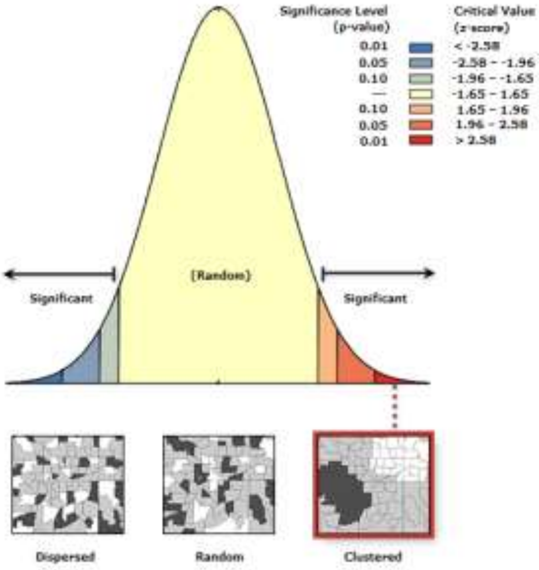

Given the z-score of 5.936701, there is a less than 1% likelihood that this clustered pattern could be the result of random chance.

Global Moran's I Summary

**Moran's Index:**	0.1331331
**Expected Index:**	-0.009709
**Variance:**	0.000564
**z-score:**	5.936701
**p-value:**	0.000000

Dataset Information

**Input Feature Class:**	Phu_yen_commune
**Input Field:**	PV_CASES_NEW.CSV.INCIDENCE
**Conceptualization:**	FIXED_DISTANCE
**Distance Method:**	EUCLIDEAN
**Row Standardization:**	False
**Distance Threshold:**	17931.5800 Meters
**Weights Matrix File:**	None
**Selection Set:**	False

Supplementary Figure 5 Trend analysis of (A) *Plasmodium falciparum* and (B) *Plasmodium vivax* by communes of Phu Yen Province, Viet Nam, 2005-2016.

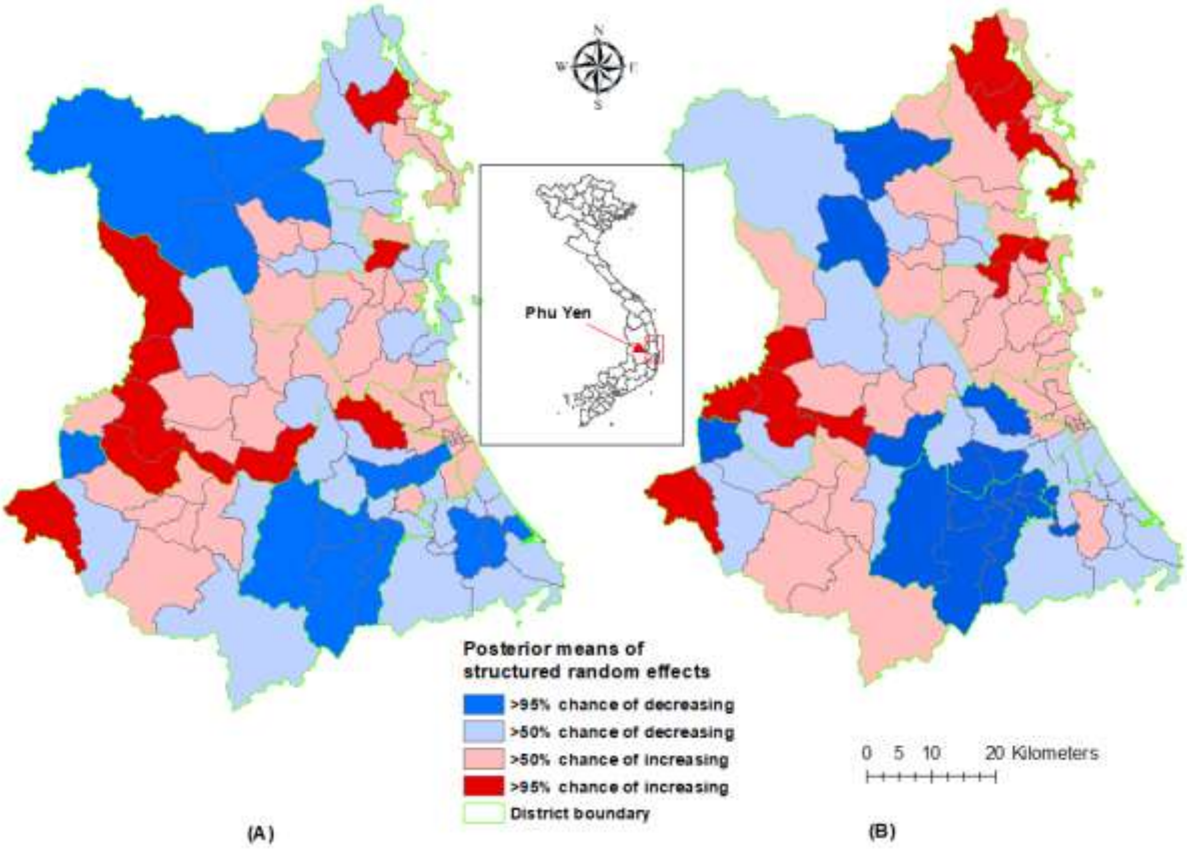

Supplementary Table 1 Independent climatic variable selection

A) Climatic variables without lag.

Variables	IRR (95% CI)	P value	AIC
Precipitation	1.004 (1.0038, 1.0044)	<0.0001	16579*
Temp max	0.88 (0.868, 0.894)	<0.0001	17110.58
Temp min	0.88 (0.863, 0.894)	<0.0001	17195.15*

* Lowest AIC, selected variable.

B) Climatic variables with one-month lag.

Variables	IRR (95% CI)	P value	AIC
Precipitation	1.004 (1.0035, 1.004)	<0.0001	16702.47
Temp max	0.998 (0.868, 0.894)	0.666	17387.48
Temp min	0.99 (0.97, 1.007)	0.231	468977.7

C) Climatic variables with two-month lag.

Variables	IRR (95% CI)	P value	AIC
Precipitation	1.002 (1.0015, 1.0021)	0.133	17245.36
Temp max	1.11 (1.096, 1.131)	<0.0001	17196.76
Temp min	1.08 (1.063, 1.105)	<0.0001	17321.29

D) Climatic variables with three-month lag.

Variables	IRR (95% CI)	P value	AIC
Precipitation	0.999 (0.9991, 0.9998)	0.002	17376.79
Temp max	1.19 (1.17, 1.21)	<0.0001	16915.03*
Temp min	1.132 (1.11, 1.155)	<0.0001	17233.34

* Lowest AIC, selected variable.

Supplementary Table 2 Tests of collinearity in the final model

Variables	VIF	1/VIF
Minimum temperature	1.01	0.994
Precipitation	1.01	0.995
Population protected	1.00	0.999
Mean VIF	1.00	

Supplementary Table 3 Model selection

A) Model comparison using Akaike's information criterion and Bayesian information criterion

**Models**	**Observations**	**AIC**	**BIC**
Poisson	14,772	23540.65	23571.05
ZIP	14,772	19573.69	19611.7

B) Results of Vuong test

Cases	Coefficient	SE	P value	95% CI
**Proportion of population protected**	**0.002**	**0.001**	**<0.0001**	**0.0012, 0.0037**
**Minimum temperature**	**0.003**	**0.0001**	**<0.0001**	**0.0028, 0.0033**
**Precipitation**	**-0.150**	**0.0095**	**<0.0001**	**-0.169, -0.132**
**Vuong test of ZIP vs standard Poisson:**			**Z= 16.8**	**Pr > z = 0.0000**
